# The Effects of Health Sector Fiscal Decentralisation on Availability, Accessibility, and Utilisation of Healthcare Services: A Panel Data Analysis

**DOI:** 10.34172/ijhpm.2021.163

**Published:** 2021-11-28

**Authors:** Arianna Rotulo, Christina Paraskevopoulou, Elias Kondilis

**Affiliations:** ^1^Global Public Health Unit, Wolfson Institute of Population Health, Queen Mary University of London, London, UK.; ^2^Faculty of Health Sciences, School of Medicine, Aristotle University of Thessaloniki, Thessaloniki, Greece.; ^3^School of Social Sciences, Panteion University of Social and Political Sciences, Athens, Greece.

**Keywords:** Fiscal Decentralisation, Decentralization, Healthcare Access, Healthcare Financing, Healthcare Equity, Geographical Disparities

## Abstract

**Background:** Fiscal decentralisation (FD) is a widely implemented decentralisation policy consisting of the allocation of pooling and spending responsibilities from the central government to lower levels of governance within a country. In 2001, The Italian National Health System (Servizio Sanitario Nazionale, SSN) has introduced a strong element of FD, making regions responsible for their own pooling of resources and for their budgets. Despite the relevance, only few studies exist on health sector-FD in Italy, mostly looking at the effects of FD on infant mortality.

**Methods:** This study performs a fixed-effects panel data analysis of Italian Regions and Autonomous provinces between the years 2001 and 2017, to investigate the effects of health sector-FD on availability, accessibility, and utilisation of healthcare services in Italy.

**Results:** FD decreases availability of staff and hospital beds, decreases utilisation of care, measured by hospitalisation rates, and increases interregional patients’ mobility for healthcare purposes, a finding suggesting increased disparities in access to healthcare. These effects seem to be stronger for public – rather than private – services, and are more prominent in poorer areas.

**Conclusion:** This evidence suggest that FD has created a fragmented and unequal healthcare system, in which levels of availability, utilisation of, and accessibility to resources – as well as the extent of public sector’s retrenchment – coincide with the wealth of the area.

## Background

**Key Messages**

** Implications for policy makers**

- Health sector fiscal decentralisation (FD) is associated with reduced availability of public healthcare staff and hospital beds.
- The degree to which FD impacts availability and utilisation of healthcare resources varies according to services’ ownership.
- Historically deprived areas experience harsher negative effects compared to wealthier areas.
- Pre-existing healthcare and socio-economic inter-regional disparities are exacerbated by FD.
- Health systems’ emergency preparedness are negatively affected by FD policies.

** Implications for the public**

Health sector fiscal decentralisation (FD) is a widely implemented health policy measure consisting in the allocation of healthcare financing responsibilities from the central government to lower levels of governance within a country. This study finds that FD is correlated with a decrease of primary, secondary and tertiary public hospital resources. At the same time, privately owned medical resources appear to increase under FD. Accordingly, accessibility and utilisation of healthcare services measured in terms of inter-regional mobility and hospitalisation rates seem to decrease both in the public and the private sphere under FD, the effect being stronger in the public sector. Areas with deficits and with a high incidence of poverty seem to experience a further exacerbated negative impact of FD on different dimensions of access to care. This factor suggests that FD may play an active role in increasing inter-regional disparities between high- and low-income groups and high- and low-income areas.

Fiscal decentralisation (FD) entails the allocation of pooling and spending responsibilities from the central government to lower levels of governance within a country.^1^ Over the years, FD and health sector decentralisation in general have gained prominence in the health policy debate as highly effective tools to achieve technical and allocative efficiency at local levels (for a comprehensive review of the theoretical literature see Rotulo et al.^1^ For an appraisal of the empirical literature on decentralisation, see the works of Abimbola et al,^2^ Dwicaksono and colleagues,^3^ and Cobos-Muñoz et al).^4^ The assumption is that when pooling happens at local levels, local actors are held more accountable for their finances, maximising the efficiency of local resources allocation.^5,6^ However, the fragmentation of the pooling system happening under FD may reduce fair redistribution (ie, based on a resources allocation formula to target population needs and demands and not based on the income and wealth of the area) of resources and foster geographical disparities.^7,8^ These contradictions are reflected in the empirical research.^9^ Studies on the impact of FD on health status carried out in high-, low-, and middle-income countries suggest that the policy is associated with a reduction of infant mortality rate, the extent of which varies according to country settings and study designs.^10-18^ However, investigations from China,^19-21^ Uganda,^22^ and Indonesia^23^ reveal that FD may decrease healthcare resources, provision, and coverage, as well as negatively affect healthcare financing and quality of care. Similarly, evidence of the effects of FD on spatial disparities suggests that FD increases inequitable access to care between the urban and the rural population as well as between residents of rich and poor areas.^14,24-27^

In the context of the coronavirus disease 2019 (COVID-19) pandemic, evidence suggest that countries with a high degree of decentralisation have adopted less stringent measures than centralised countries.^28-30^

Italy has a long history of inter-regional socio-economic disparities, reflected in a clear-cut divide between the rich, industrialised areas of the Centre-North, and the poorer regions in the South. The Italian National Health Service (Servizio Sanitario Nazionale, henceforth SSN) was established to guarantee equal and uniform access to care irrespective of income or location, based on the principle of solidarity, in the attempt to reduce some regional differences. To facilitate this aim, the SSN was financed through general taxation at the central level, while planning and allocation of resources occurred at the local levels.^31^ Over the years, the SSN has experienced a strong decentralisation process, culminating in 2001 with a Constitutional reform enacting health sector FD.^32^ The national health system was restructured into several regional services, each one responsible not only for planning of services and allocation of resources, but also for their financing.^33,34^ Although the central government has kept funding responsibilities for some *essential levels of care* (Livelli Essenziali di Assistenza), all other activities were funded by the regional healthcare budget, financed by (*i*) two newly introduced regional taxes, (*ii*) patients’ cost-sharing mechanisms, and (*iii*) fees for carrying out private practices in public facilities (known as intramoenia activity).^32-34^ To expand their budgets, regions acquired the power to introduce, increase, or reduce the share of regional taxes to be levied. They could also determine the amount of co-payments for public provision. Moreover, local authorities became free to outsource the provision of services to private accredited firms, or to directly reimburse private providers for the provision of the *essential levels of care,* where regional system’s capacity was insufficient.^35,36^ Similarly, both public and private accredited providers could accept patients from different regions and get reimbursed by the region of origin, based on a system of regional diagnostic related groups.^33,34,37^ Accordingly, local authorities were also held accountable for any profit or deficit they generated.^31,33,34^

Despite a strong element of FD embedded to the Italian SSN, only few studies have investigated the effects of health sector-FD in Italy – mainly on infant mortality rate,^16,38^ and on self-assessed health status.^39^ Like the Italian literature, also the international evidence on health sector-FD have focused on the health outcomes dimension only or on the practice of fiscal federalism.^1^ This study builds on existing knowledge and presents a multidimensional analysis of the impact of health sector-FD on three dimensions of access to care – availability, accessibility, and utilisation of healthcare services – in Italian regions between 2001-2017. The contributions to the existing literature are many: first, this study provides an empirical assessment of the effects of FD on multiple dimensions of healthcare services, rather than on one dimension only. In particular, it investigates the relationship between FD and (*i*) the availability of non-financial healthcare resources, (*ii*) the extent to which the services are reachable, and (*iii*) the extent to which the services are used. Second, it furnishes an assessment of FD from a healthcare system standpoint, rather than from a health outcomes perspective. Third, to the authors best knowledge this is the first attempt at making such a detailed empirical analysis. Past studies, in fact, have either investigated FD from a health status perspective (see, for example, Cavalieri and colleagues, and Di Novi et al^16,38,39^), from a healthcare financing perspective, or from a not-healthcare-related standpoint.

## Methods

###  Data and Variables

This study employs a panel of 19 Italian regions plus the two Autonomous Provinces Trento and Bolzano (Trentin/Sud-Tyrol region) over the period 2001-2017. Differently from other provinces, Trento and Bolzano share the same political, administrative, and legislative competences of Italian regions, despite being provinces of the Trentin/Sud-Tyrol region, as well as the same autonomy of Special Statute Regions.^40^ Accordingly, relevant fiscal data for Trentin/Sud-Tyrol region are available at the provincial level only. Differently from Ordinary Statute Regions, Special Statute Regions and Autonomous Provinces have benefitted from a not-health specific decentralised autonomy over the years. However, when the FD reform was implemented, all regions gained the same responsibilities and autonomies with regards to health and healthcare services.

FD is defined as the shift of spending authority and pooling responsibility from the central government to local levels within a country.^7^ The FD indicator employed in this study (FDHE – Fiscal Decentralisation of Health Expenditure) is measured as the ratio of local public health expenditure to total public health expenditure. More precisely, the numerator is regional health expenditure, while the denominator is the sum of regional health expenditure and central health expenditure for each region. Fiscal data have been collected from Territorial Public Accounts (TPA) ^[1]^.^41^ Differently from an analogous study that structured health sector-FD indicator using the share of local not health-related revenues to total revenues,^16^ this model does not employ a measure of decentralisation from the revenue-side. The choice is guided by the fact that regional revenues figures from TPA are not health-specific, hence do not provide an adequate snapshot of FD in healthcare.^41^ On the other hand, the sector specificity of TPA’s expenditure data is exploited to compute a health-specific FD indicator that can reflect the changes in regional healthcare financing following the 2001 FD reform.

Access to care relates to how many people can use a healthcare service and reflect patients’ ability to enter the system.^42,43^ Although access is considered a general concept, it encompasses a series of intertwined dimensions that determine the degree to which patients gains access to care.^44^ To evaluate FD, this work employs three of these dimensions: the volume of available resources (availability); the ability of patients to access the service (accessibility); and the level of use of accessible services (utilisation).^43-46^ Variables have been drawn from the Health for All database, available from the Italian Statistical Bureau website.^47^ Types and number of indicators for each dimension have been chosen according to whether data were available for the whole time-series and cross-sections.

 Availability of services is measured in terms of the density of human resources and technical resources. Respectively, the human resources indicator consists of the density of general practitioners (GPs) per 10 000 population (GP_DENSITY) and density of public and private healthcare staff, divided by total staff, doctors, and nurses at the primary, secondary, and tertiary level of care (ALLSTAFF_PUBPRIV; ALLSTAFF_PUB; ALLSTAFF_PRIV; DOCTORS_PUBPRIV; DOCTORS_PUB; DOCTORS_PRIV; NURSES_PUBPRIV; NURSES_PUB; NURSES_PRIV). Technical resources are expressed in terms of density of total and acute public and private secondary and tertiary hospital beds per 10 000 population (TOT_BEDS; TOT_NHS_BEDS; TOT_PRIV_BEDS; TOT_ACUTE_BEDS; PUB_BEDS_ACUTE; PRIV_BEDS_ACUTE) and density of medical machinery – such as magnetic resonance imaging (MRI) scanners, computerized tomography (CT) scanners (CT_DENSITY), haemodialysis machines (HEMOD), and operating tables (OPERATING TABLE) – per 10 000 population at the secondary and tertiary level.

We accept that the features of FD may limit the redistributive effect of a central pool.^7,48^ Regions may thus adopt strategies to decrease their local public expenditure to match locally available resources.^5^ Accordingly, it is expected that FD overall reduces the availability of healthcare services.

To overcome the lack of direct measures for accessibility of care (eg, waiting lists), the study employs the percentage of hospitalised patients residing in the same region where the hospitalisation event occurred (RESIDENT_PATIENTS) as an indicator to estimate potential geographical barriers to access. Such choice implies that one opts for treatment outside the region of residence because they expect to receive a service of better quality than in their regions of residence or because waiting times to obtain medical services are shorter.^49,50^ Granted that FD encourages patients’ interregional flow,^51^ especially from regions with historically weaker healthcare services, it is expected to see a decrease in the percentage of hospitalised resident patients, confirming the increased practice of regional mobility.^49,52^

Lastly, to quantify utilisation of care, the model employs total and acute hospitalisation rates in public and private hospitals (TOT_HOSP_RATE; TOT_HOSP_RATE_ACUTE; PUB_HOSP_RATE; PUB_HOSP_RATE_ACUTE; PRIV_HOSP_RATE; PRIV_HOSP_RATE_ACUTE). Hospitalisation rates are measured using hospital discharges – that is, the number of cared patients leaving the hospital after having spent at least one night.^53^ Although the discharge event includes both alive and dead patients, the number of hospital discharges equals the number of hospital admission and in this research the indicator is used as a measure of hospital services utilisation rather than as a measure of health outcomes and hospital performance.^47,53^ We assume that under FD regions try to reduce their public health expenditure by – among other things – limiting the number of hospital admissions. Therefore, we expect that the FDHE indicator decreases discharge rates.

The study also includes some *fiscal disparities* variables, to control for differences in terms of own local revenues (OWN_REVENUES), fiscal imbalance (TOT_SURPLUS), relative poverty (RELATIVE_POVERTY) and per capita gross domestic product (GDP) (PC_GDP) at the regional level. Regional revenues measure how much of the total regional income comes from regionally raised revenues. The indicator is available from TPA.^41^ Fiscal imbalance is calculated by subtracting total regional expenditure from total regional revenues. Both indexes come from TPA.^41^ Relative poverty measures the share of regional population living in relative poverty, and per capita GDP measures the level of regional GDP per capita. Both indicators have been selected from the Health for All database.^47^ Descriptive statistics for the employed variables are presented in Table 1.

**Table 1:** Descriptive Statistics for Availability Variables

**Variable**	**Mean**	**Median**	**Maximum**	**Minimum**	**SD**	**Obs.**
Primary and secondary care (availability)						
GP_DENSITY Density of general practitioners per 10 000 population	7.96	8.11	9.37	4.98	0.81	357
TOT_BEDS Density of hospital beds per 10 000 population	37.35	36.90	54.69	20.87	5.84	357
TOT_NHS_BEDS Density of public hospital beds per 10 000 population	30.99	31.37	47.82	14.68	5.95	357
TOT_PRIV_BEDS Density of private hospital beds per 10 000 population	6.36	6.25	18.04	0.00	3.60	357
TOT_ACUTE_BEDS Density of acute hospital beds per 10 000 population	32.33	31.82	46.73	17.61	5.27	357
PUB_BEDS_ACUTE Density of public acute hospital beds per 10 000 population	28.48	28.33	46.40	14.12	5.55	357
PRIV_BEDS_ACUTE Density of private acute hospital beds per 10 000 population	4.10	3.98	12.25	0.00	2.61	323
Human and technical resources (availability)						
ALLSTAFF_PUBPRIV Density of healthcare workers per 10 000 population	107.41	105.57	175.76	70.32	17.20	357
ALLSTAFF_PUB Density of public healthcare workers per 10 000 population	96.98	97.73	168.25	53.86	17.61	357
ALLSTAFF_PRIV Density of private healthcare workers per 10 000 population	10.43	10.10	24.04	0.00	5.67	357
DOCTORS_PUBPRIV Density of doctors per 10.000 population	20.32	20.53	28.03	14.20	2.60	357
DOCTORS_PUB Density of publicly employed doctors per 10 000 population	17.86	17.45	25.93	12.18	2.29	357
DOCTORS_PRIV Density of privately employed doctors per 10 000 population	2.46	2.35	5.68	0.00	1.41	357
NURSES_PUBPRIV Density of nurses per 10.000 population	46.00	46.83	62.64	30.98	6.38	357
NURSES_PUB Density of publicly employed nurses per 10 000 population	42.90	43.56	60.60	25.83	6.76	357
NURSES_PRIV density of privately employed nurses per 10 000 population	3.10	2.79	7.13	0.00	1.84	357
MRI Density of MRI scanners per 10.000 population	0.13	0.13	0.32	0.017	0.05	315
HEMOD Density of haemodialysis machines per 10 000 population	2.57	2.55	5.11	0.75	0.83	315
CT_DENSITY Density of CT scans per 10 000 population	0.23	0.22	0.45	0.08	0.06	357
OPERATING_TABLE Density of operating tables per 10 000 population	1.49	1.42	3.42	0.86	0.33	315
Accessibility variable						
RESIDENT_PATIENTS Percentage of patients hospitalised in their region of residence	80.17	80.94	95.99	25.66	6.82	357
Utilisation variables						
TOT_HOSP_RATE Total hospitalisation rate per 10 000 population	1338.59	1326.00	2020.60	795.80	216.27	357
PUBLIC_HOSP_RATE Hospitalisation rate in a public facility per 10 000 population	1174.78	1162.50	1880.50	654.70	212.21	357
PRIV_HOSP_RATE Hospitalisation rate in a private facility per 10 000 population	163.81	141.10	536.80	0.00	107.47	357
TOT_HOSP_RATE_ACUTE Total hospitalisation rate for acute care per 10 000 population	1280.47	1258.70	1991.30	758.20	217.67	357
PUB_HOSP_RATE_ACUTE Hospitalisation rate for acute care in a public facility per 10.000 population	114.57	113.60	185.10	64.70	21.17	357
PRIV_HOSP_RATE_ACUTE Hospitalisation rate for acute care in a private facility per 10 000 population	134.83	106.20	439.20	0.00	102.34	357
Independent and fiscal disparity variables						
FDHE Share of regional health expenditure to total regional health expenditure	0.9914	0.9900	1	0.9300	0.0102	288
RELATIVE_POVERTY Incidence of households living in relative poverty	11.56	8.79	30.97	1.23	7.48	288
LN_OWN_REVENUES Logarithm of regional revenues coming from own regional taxes	7.15	7.09	9.98	3.65	1.17	357
LN_PC_GDP Logarithm of regional GDP per capita	10.08	10.14	10.62	9.53	0.27	315
LN_TOT_SURPLUS Logarithm of fiscal imbalance: total regional revenues minus total regional expenditure	6.73	6.83	9.20	3.55	1.03	296

Abbreviations: SD, standard deviation; MRI, magnetic resonance imaging; HEMOD, haemodialysis machines; FDHE, fiscal decentralisation of health expenditure; CT, computerized tomography; GDP, gross domestic product; GP, general practitioner; NHS, National Health Service.

###  Methods and Robustness Tests

 The analysis is based on the following model:


$$Y_{it}=\beta_{i,0}+\beta_{i,j}X_{i,t}+u_{i}$$

where i=1….21, different regions/autonomous provinces, *t* is the time, and *X*is the set of covariates under investigation. *β*_i,0_ is the country fixed-effects that expresses the region-specific patterns and time-invariant determinants of the FD process.

To assess the robustness of the model, summary unit root tests including Levin, Lin & Chu test, Breitung test, Im, Pesaran, Shin test, augmented Dickey-Fueller test, and Fisher test^54-57^ are performed, to verify that all variables are non-stationary in levels (*P* > .1) and stationary in first difference (*P* < .01). Stationary variables in levels are not suitable for the purpose of this analysis as they lead to spurious results, that is significant results from unrelated data. On the other hand, stationarity in first difference produce more reliable data, since the series is stripped of its trend and seasonality.

The second stage of the analysis includes ordinary least squares (OLS) panel regressions with fixed effects, between FDHE and the employed variables corrected for heteroskedasticity. The Hausman specification test determined the appropriateness of the fixed-effects model to control for region-specific, time-invariant characteristics.^58^ The result is in line with expectations, as fixed-effects are employed when variables are constant across individuals and change at a constant rate over time and thus have a fixed effect over time.

Lastly, based on the assumption that the impact of a new policy on outcome variables takes a varying period of time before it actually occurs a vector auto-regressive model is performed, and the Akaike information criteria is employed to estimate the optimal lag length for the model, so to have a more precise estimate of the effects of FDHE on all variables.^59^ Based on the results of vector auto-regressive and Akaike information criteria, the study opts for a five-years lag for all the dependent variables. Although lagged variables are a well-known appropriate tool to control for potential endogeneity,^38^ the study resorts to an additional robustness test based on system generalised method of moment (SGMM) with instrumental variables (IV) and an exogenous variable based on kilometres of coastal lands, to check for potential endogeneity. Although SGMM and IV are considered useful strategies to produce estimates that are not plagued by endogeneity, their use comes with a series of more severe limitations. The asymptotic properties of SGMM and IV cannot be satisfied by the small sample employed in this research (ie, 21 cross-sections). With a sample outstripped of its statistical power, the risk of generating even more biased estimations than the endogenous ones would be is high.^38,60^

 For this reason SGMM is not employed as the main strategy but as an additional robustness check whose results (in line with the main estimates of the employed model) are available upon request.

To further increase the reliability of the employed model and effectively rule out the presence of endogeneity in the employed model, this study also resorts to an autocorrelation test of the residuals of each independent variable for each original static regression. Results (available upon request) suggest that the majority of OLS estimations do not present endogeneity. When present, an additional OLS has been run without the endogenous variable and results have been consistent with our initial observations, suggesting that this study’s estimations are not plagued endogeneity. OLS results are displayed in Tables 2, 3, and 4.

## Results

###  Health Sector-FD in Italy and its Effect on Availability of Care 

FD estimates produced by the FDHE index show that over the years health sector-FD has maintained a steady upgoing trend (Figure), despite suffering some setbacks in 2004 and 2011.

**Figure:**
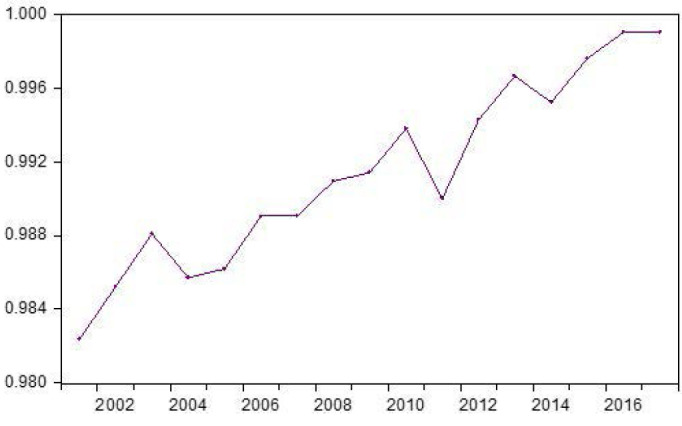
Mean of Fiscal Decentralisation Index, 2001-2017, Italy, Based on Data From Territorial Public Account’s.^41^

Tables 2, 3, and 4 show the fixed-effects estimations for availability, accessibility and utilisation of healthcare services, whereas Table 5 reports the absolute impact of FDHE and of the fiscal disparity variables on the three dimensions of interest. The output in Table 2 indicates that the effect of FD (measured as the ratio of regional public health expenditure to total regional public health expenditure) on availability indicators for human resources is – in most of the cases – negative and significant with a high goodness of fit. More specifically, we find that a unit increase of FD of health expenditure results in a 0.09 reduction in the number of GPs per 10 000 population. Results further indicate that one unit increase in FDHE is associated with a reduction of density of all healthcare staff by 0.98 and of public healthcare staff by 0.89, which implies an absolute decrease by 0.017 and 0.015, respectively. The effects are significant at the 1% with a high goodness of fit. Differently, the density of private healthcare staff seems to increase under FD, although the effect is not statistically significant. FDHE also appears to have a positive but not significant relationship with density of total and public doctors, while the density of private doctors appears to increase by 0.11 and remains statistically significant. The incidence of households living in relative poverty is also associated to a slight increase of private doctors’ density by 0.02 (*P*< .01) and to a decrease in public doctors by 0.04 (*P*< .05). Relative poverty is also correlated to a minor decrease of the number of public healthcare staff by 0.18, significant at the 10% level. The impact of FDHE on density of nurses is similar to that of all healthcare staff: a unit increase in our FD indicator leads to a 0.51 and 0.50 decrease in the total number of nurses and in the number of public nurses per 10 000 population respectively. Both outcomes are significant at the 1%. Over time, the absolute effect of FD is -0.009 and -0.008, respectively. Density of private nurses appears to increase under FD by 0.04, the effect being significant at the 5% level. Overall, the analysis suggests that FD has a negative impact on human resources, with the density of all type of staff and nurses decreasing for each unit increase of FD of healthcare spending.

**Table 2:** Ordinary Least Squares Regressions of Human Resources Variables (Availability)

**Variables**	**GP_Density**	**All Staff PubPriv**	**All Staff Pub**	**All Staff Priv**	**Doctors PubPriv**	**Doctors Pub**	**Doctors Priv**	**Nurses PubPriv**	**Nurses Pub**	**Nurses Priv**
FDHE	-0.09**	-0.98***	-0.89***	0.11	0.15	0.05	0.11***	-0.51***	-0.50***	0.04**
LN_OWN_REVENUES	0.20***	1.16	0.85	0.13	-0.24	-0.47	0.08	1.72***	1.64***	0.01
LN_TOT_SURPLUS	-0.05***	-0.73**	-0.68**	-0.04	0.06	0.08	-0.003	-0.38*	-0.40**	-0.01
RELATIVE_POVERTY	-0.004	-0.08	-0.18*	0.01	-0.001	-0.04**	0.02***	0.08	0.04	0.01***
LN_PC_GDP	-0.07	0.59	-6.72	1.64***	2.22***	1.23***	0.59***	-1.15	-2.56	0.42***
Adj. R^2^	0.95	0.95	0.96	0.98	0.91	0.87	0.97	0.93	0.94	0.98

Abbreviations: FDHE, fiscal decentralisation of health expenditure; GDP, gross domestic product; GP, general practitioner. *** *P* < .01, ** *P* < .05, * *P* < .1.

Table 3 presents the impact of FDHE on technical resources. Estimations suggest that FD decreases the density of total, public, and private hospital beds by 3.16, 2.88, and 0.23, respectively, other things being equal. The overall decrease of hospital beds is by -0.053, -0.049, and -0.004, respectively. The effect is consistent also for the density of hospital beds for acute care. Further, FDHE is correlated to a reduction of acute beds by 3.48 (with an absolute effect of -0.059), and in particular of public and private acute beds by 2.90 and 0.32 respectively (with an absolute effect of -0.049 and -0.005, respectively). It is worth noting that the incidence of households living in relative poverty is negatively correlated to the density of hospital beds, suggesting an inequitable distribution of secondary services. The estimation in Table 3 also highlights that regional surpluses play a negative effect on hospital beds density. Thus, suggesting that regions with more fiscal space do not necessarily invest more in hospital resources. Contrarily to hospital beds variables, the density of medical devices seems to be positively correlated to FD. In particular, an increase of FDHE increments the number of MRI machines, haemodialysis machines, and CT scanners per 10 000 population by 0.02, 0.10, and 0.007, respectively, with an absolute impact of 0.0003, 0.002, and 0, respectively. These values are all significant at the 1% level (MRI) and 5% level (haemodialysis and CT scanners), with a goodness of fit above 60%. On the opposite, FD is associated with a decrease in the density of operating tables, although the impact is not statistically significant. Instead, the density of MRI machines, CT scanners, and haemodialysis show a mild positive and statistically significant (*P*< .01) correlation with the amount of regional wealth.

**Table 3:** Ordinary Least Squares Regressions of Technical Resources Variables (Availability)

**Variables**	**tot_beds**	**tot_nhs_beds**	**tot_priv_beds**	**tot_acute_beds**	**pub_beds_acute**	**priv_beds_acute**	**MRI**	**Hemodyalisis**	**CT Scanners**	**Operating Tables**
FDHE	-3.16***	-2.88***	-0.23***	-3.48***	-2.90***	-0.32 ***	0.02***	0.10***	0.007***	-0.005
LN_OWN_REVENUES	1.24	1.58**	0.23	1.67*	1.45*	0.06	0.002	-0.07	-0.008	0.05
LN_TOT_SURPLUS	-0.57***	-0.52**	-0.06*	-0.76***	-0.55**	-0.06**	-0.00002	0.01	0.002	-0.01
RELATIVE_POVERTY	-0.22***	-0.24***	-0.03***	-0.41***	-0.30***	-0.04***	0.0008	0.006	0.0004	-0.003
LN_PC_GDP	-1.33	-5.81	1.92***	-4.87***	-7.88	-0.29	0.12**	0.98***	0.10***	-0.12
Adj. R^2^	0.73	0.82	0.98	0.65	0.77	0.98	0.7	0.96	0.8	0.93

Abbreviations: MRI, magnetic resonance imaging; FDHE, fiscal decentralisation of health expenditure; GDP, gross domestic product. *** *P* < .01, ** *P* < .05, * *P* < .1.

**Table 4:** Ordinary Least Squares Regressions of Accessibility and Utilisation Indicators

**Variables**	**Resident Patients**	**Tot hosp Rate**	**Public hosp Rate**	**Private hosp Rate**	**Tot hosp Rate Acute**	**Public hosp Rate Acute**	**Private hosp Rate Acute**
FDHE	-0.29***	-96.43***	-81.15***	-8.34***	-101.85 ***	-83.11***	-10.07***
LN_OWN_REVENUES	0.64*	67.65*	46.11	13.35*	67.72*	43.53	10.34**
LN_TOT_SURPLUS	-0.16**	-10.41	-8.87	-2.79	-12.42	-8.59	-2.42
RELATIVE_POVERTY	-0.06*	-8.45*	-6.21	-0.84	-9.94*	-6.81	-1.17*
LN_PC_GDP	-0.66	-90.02	-165.44	22.34	-159.32	-187.03	5.32
Adj. R^2^	0.99	0.82	0.72	0.96	0.78	0.71	0.97

Abbreviations: FDHE, fiscal decentralisation of health expenditure; GDP, gross domestic product. *** *P* < .01, ** *P* < .05, * *P* < .1.

**Table 5:** Absolute Impact of FDHE and of the Fiscal Disparities Variables on Availability, Accessibility, and Utilisation of Healthcare

**Variables**	**FDHE**	**OWN_REVENUES**	**TOT_SURPLUS**	**RELATIVE_POVERTY**	**PC_GDP**
GP_density	-0.002	0.003	-0.005	0.000	-0.001
All staff PubPriv	-0.017	0.017	-0.070	-0.003	0.012
All staff Pub	-0.015	0.012	-0.065	-0.007	-0.138
All staff Priv	0.002	0.002	-0.004	0.000	0.034
Doctors PubPriv	0.003	-0.003	0.006	0.000	0.046
Doctors Pub	0.001	-0.007	0.008	-0.002	0.025
Doctors Priv	0.002	0.001	0.000	0.001	0.012
Nurses PubPriv	-0.009	0.025	-0.036	0.003	-0.024
Nurses Pub	-0.008	0.023	-0.038	0.002	-0.053
Nurses Priv	0.001	0.000	-0.001	0.0004	0.009
tot_beds	-0.053	0.018	-0.054	-0.009	-0.027
tot_nhs_beds	-0.049	0.023	-0.050	-0.010	-0.119
tot_priv_beds	-0.004	0.003	-0.006	-0.001	0.039
tot_acute_beds	-0.059	0.024	-0.073	-0.016	-0.100
pub_beds_acute	-0.049	0.021	-0.053	-0.012	-0.162
priv_beds_acute	-0.005	0.001	-0.006	-0.002	-0.006
MRI	0.0003	0.000	0.000	0.000	0.002
Hemodyalisis	0.002	-0.001	0.001	0.000	0.020
CT scanners	0.000	0.000	0.000	0.000	0.002
Operating tables	0.000	0.001	-0.001	0.000	-0.002
Resident Patients	-0.005	-0.011	0.009	-0.002	-0.074
Tot hosp rate	-1.629	0.964	-0.994	-0.339	-1.852
Public hosp rate	-1.371	0.657	-0.847	-0.249	-3.403
Private hosp rate	-0.141	0.190	-0.266	-0.034	0.459
Tot hosp rate acute	-1.721	0.965	-1.186	-0.398	-3.277
Public hosp rate acute	-1.404	0.620	-0.820	-0.273	-3.847
Private hosp rate acute	-0.170	0.147	-0.231	-0.047	0.109

Abbreviations: MRI, magnetic resonance imaging; FDHE, fiscal decentralisation of health expenditure; CT, computerized tomography; GDP, gross domestic product; GP, general practitioner.

###  The Effects of Health Sector-FD on Accessibility and Utilisation of Services 

Table 4 shows the impact of FD on accessibility of services (resident patients) and utilisation of services (hospitalisation rate). Outputs show that an increase of FD decreases resident hospital patients by 0.29% with an absolute change by -0.005, suggesting an increase in the share of patients moving from one region to another to seek treatment. The result is significant at the 1% level. In line with the decrease of hospital beds showed in Table 1, the model shows that FD has a negative association to rates of hospitalisation at all levels. In particular, one unit increase of FD decreases the total hospitalisation rate and total hospitalisation rate for acute care by 67.65 (with an absolute decrease by 1.63) and 67.72 (with an absolute decrease by 1.72) respectively per 10 000 population. Similarly, total public and private hospitalisation rates drop by 81.15 and 8.34 respectively (with an absolute decrease by 1.37 and 1.40, respectively), while acute public and private hospitalisation rates decrease by 83.11 and 10.07 with an absolute decrease by 1.40 and by 0.17). All effects are significant at the 1% level, and present a high goodness of fit, with R2 ranging between 72% and 97%. Interestingly, results further show that an increase in own regional revenues have a moderate role in increasing hospitalisation rates, while relative poverty correlates with a moderate reduction of admission rates.

## Discussion

The aim of this study was to analyse the effect of health sector FD on three dimensions of access to care – availability, accessibility, and utilisation of healthcare services – in Italian regions between 2001-2017. The overall results presented in Tables 2, 3, and 4 offer a snapshot of the disadvantageous impact of FD on the Italian healthcare system. In particular, the model shows that FD has a negative impact on the density of human and hospital resources. Accordingly, FD seems to be correlated to a general fall in hospitalisation rates as well as to reduced accessibility of services, signalling that the percentage of hospitalised resident patients to total hospitalised patients decrease under FD. It is also worth noting, however, that a general fall in hospitalisation rates may be linked to increased accessibility to preventative care, although our model suggests a decrease in the number of GPs after FD.

Over the years, FD has become an ever more prominent feature of the Italian health system. Figure shows that health sector-FD in Italy has expanded over years, despite some fluctuations. The downward slope occurring in 2004, for example, is likely associated with the high deficits some regions generated, which required a bail-out from the national government and the enforcement of *recovery plans*, structural adjustments at local levels to achieve national macroeconomic objectives.^31,61^ Similarly, the downward trend in 2009 suggests limited fiscal space for healthcare at regional levels coinciding with austerity policies and expenditure cuts implemented all through the great recession.^31^

Results’ general trend confirms the theoretical assumption that FD sits within a wide group of cost-containment and market-driven strategies.^5,6,62^ Indeed the implementation of FD operates as a cost-control mechanism at regional levels to facilitate national budget-cutting objectives.^63^ In fact, the evidence on availability indicators (Tables 1 and 2) reveal that FD in Italy coincides with a change of priorities for regional resources’ allocation: from staff and hospital beds to *physical capital* – ie, medical devices. This is a seemingly contradictory finding, since healthcare systems are *stagnant economic sectors*, that is, economic systems in which technology does not replace labour. On the contrary, increased availability of medical technology creates the need for new medical specialties and for more healthcare workers.^64^ this contradiction could be explained by the decision by regions with high medical devices’ obsolescence to invest high shares of their budgets on new equipment to increase access to medical technology, at the expenses of other resources.^31,65,66^ This shift from hospital investment to medical technology may potentially reduce occurrence of more serious health problems in certain areas. Another explanation could be that these contradictory findings are merely a reflection of contradicting priorities between different actors within the healthcare system; increased private investment on profitable medical technology and coexisting public disinvestment from hospital care and healthcare workers due to austerity measures, especially during the Great Recession.^61,67^

Similarly, the scarcity of regional financial resources for the training of new GPs to replace retiring ones explains the reduction of primary care physicians.^36,68^ Austerity measures during the Great Recession may have contributed to further lower availability.^68^ On the other hand, Evidence of lower utilisation rates and increased inter-regional healthcare mobility confirm that all through the expansion of health sector-FD, regional healthcare services have been scaled-down in a way that has affected the local supply of care. This finding is supported by previous empirical evidence of diverted local funding,^22^ deteriorating preventative services,^19^ and decreased availability and utilisation of services under FD.^20^

Additionally, estimates presented in Tables 1 and 2 show that the degree to which FD impacts on availability and utilisation indicators varies according to services’ ownership. In particular, FD is negatively correlated to the density of public sector’s human resources, while it seems to bear a positive effect (although not statistically significant) on private sector’s. Similarly, the negative impact of FD on availability of hospital beds and on hospitalisation rates is much more evident in public settings than in private ones. The fact that private providers are less affected by local budget cuts – since they can rely on alternative sources of profit (eg, private contributions) – furnishes an initial explanation to the result. The gradual expansion of the private sector within regional services is another factor to account for.^35,69^ Different results according to services’ ownership, in fact, suggest that FD may have facilitated the retrenchment of the public sector at regional levels. Indeed, the new financing framework introduced by FD encouraged regions to adopt – to varying extents – market-based approaches for healthcare delivery, mostly consisting of a public-private mix, confirming that FD may serve as an alternative path to outsource.^62^ Lombardy, for example, opted for a private-oriented organisation of services, financed through taxes and high co-payments and provided almost entirely by the private sector.^35^ Similarly, regions with high deficits and historically weaker healthcare systems have been increasingly contracted-out services to private providers.

The outputs produced by the fiscal disparity variables (Tables 2, 3, and 4) suggest that FD perpetuates and at any rate widens pre-existing spatial disparities between regions, in line with the observations of Lago et al,^70^ Sanogo,^24^ Hodge et al,^25^ but in contrast with the findings of Di Novi et al,^39^ and Cavalieri et al.^16,38^

Regional surpluses represent the relationship between expenditure and revenues. Increased surplus may either be related to high income levels for a given level of expenditure or to lower expenditure levels for a given level of income. In other words, high surpluses could be encountered both in areas with a higher degree of austerity (where the level of expenditure is kept below the level of revenues) and in areas that by implementing austere measures become wealthier. The findings, thus, suggest that higher surpluses (eg, austerity) are related with a statistically significant decrease of all public healthcare workers and especially of public nurses (see Table 2, row 5, columns 3 and 10).

Relative poverty is defined as the increased incidence of households living below the poverty threshold. This could be the case either in a poor region or in a relatively wealthy area with high socio-economic inequalities. The findings suggest that relative poverty is related with a decrease of all public healthcare workers, especially doctors, and with a significant increase of private doctors and nurses (see Table 2, row 5, columns 4, and 7-10). This seemingly contradictory finding could be explained by either the level of wealth of a region or by high socio-economic inequalities. Relative poverty also decreases total acute and ordinary hospitalisation rates, especially in the private sector, which might be a sign either of better or unmet healthcare needs (see Table 3, row 5, columns 2-8).

 Per capita GDP is a measure of the average wealth of a region, without necessarily taking into consideration the level of income distribution and inequality. Findings suggest that the higher the average per capita GDP, the higher the provision of all private healthcare resources (doctors, nurses, and hospital beds) and the higher the availability of public doctors. This is an indirect sign that the private sector follows the demand, therefore the income. All the above suggest that in poor regions, in regions with high inequalities, and in regions with austerity measures in place there are signs of low provision of public services, unmet hospital needs and decreased demand – and therefore provision – of private services, whose access is based on the level of income.

Low availability and utilisation bear direct implications on accessibility to care. To meet their needs, patients either engage with the private sector or travel to neighbouring regions for treatment, as the evidence of increased patients’ inflows indicates.^49^ The accessibility indicator (resident_patients), in fact, shows that all through FD the share of residents to total patients accessing regional acute services lowers, suggesting increased inter-regional healthcare mobility for acute hospital treatment. Reduced availability of public resources in some regions and the increased prominence of the private sector are barriers to access that account for this trend. Another hypothesis is that higher hospitalisation rates of *foreign* patients are the result of supplier-induced demand at hospital levels, to maximise reimbursement rates from the region of origin. The consequence, in any case, is the diversion of funds destined to local acute healthcare services from poorer regions to areas with stronger services—yet another way of perpetuating inter-regional inequities. However, further research to understand the impact of FD on inter-regional mobility and the relative movement of capital attached to that is required.

The outcomes of FD on regional healthcare services highlighted by this investigation have direct policy implications on the level of emergency preparedness in the Italian SSN and – more in general – in decentralised health systems, as suggested by recent observations.^28-30^ Preparedness concerns the ability to anticipate, respond to, and recover from the impacts of an emergency. A health system with adequate levels of trained staff and hospital resources lies at the foundation of successful preparedness, since it can guarantee continuity of care while addressing the emergency.^71,72^ Italy’s capacity to effectively manage and control the COVID-19 epidemic in 2020 suffered from low availability of resources, fragmentation of services, weak public provision, and spatial disparities, factors that FD has exacerbated and that have played a role in the late epidemic response of the country.^73^

###  Study Limitations 

Notwithstanding the efforts to ensure a rigorous application of research methods, this study bears some weaknesses. The limited availability of uninterrupted time series informed the choice of our indicators, which thus provide an indirect measurement of the three dimensions we aimed to explore. Further to that, the lack of an indicator measuring the volume of regional revenues devoted to healthcare guided our decision to structure our FD indicator using health expenditure data, rather than a combination of total revenue estimates that could not account for the healthcare portion. Although a similar measurement has been widely used in pre-existing FD literature, it is important to consider that the indicator may suffer from endogeneity issues, as mentioned by Fisman and Gatti.^74^ Lack of data availability on the ownership status of medical equipment (private for profit or public) poses a further limitation to the interpretation of our findings.

## Conclusion

 This research has produced a detailed, multidimensional empirical analysis, the breadth of which is still lacking in the field of health sector FD research. Although it is accepted that FD is a policy option used to achieve local cost-efficiency, it risks increasing inter-regional disparities and inequities between high-and-low-income groups. The general trend emerging from the case of Italy reveals that FD has a negative effect on the availability of human and hospital resources.FD also seems to reduce utilisation of healthcare services and accessibility thereof, signalled by reduced hospitalisation rates and increased interregional mobility for acute hospital care, respectively. Interestingly, the effects of FD also come abreast of low provision of public services and unmet hospital needs in poorer areas and areas with high austerity and/or disparities. Our contribution confirms the hypothesis that FD may contribute to widen the gap between different income groups, by making rich regions (and wealthy population) better-off and poor regions (and poorer population) worse-off.

 Contributions to the field are still needed and further research should be strongly encouraged. In particular, future efforts should focus on producing more generalisable evidence on the effects of FD on national health systems. An option could be to perform a comparative study between countries with similar public health services and similar levels of FD in place. Within the context of Italy, further efforts should be directed at exploring the role of FD and of different institutional setting and of local health policies on regional healthcare systems. This could be done through a policy analysis and an operationalisation of different regional policies and models. Alternatively, future investigations should appraise how the impact of FD changes in high- and low-income regions, and whether inter-regional mobility occurs for preventative or curative purposes. After having appraised the correlation between FD and different dimensions of healthcare provision, there is indeed the need to simulate and predict the effects of a decrease of FD. Furthermore, future efforts should be focused in appraising the extent to which geographical variations of hospital discharges reflect regional differences on the epidemiological distribution of morbidity.

## Ethical issues

 Not applicable: the study employed secondary data for which ethical approval was not applicable.

## Competing interests

 Authors declare that they have no competing interests.

## Authors’ contributions

 Conception and Design: AR, CP, and EK. Acquisition of data: AR. Analysis and interpretation of data: AR and CP. Drafting of the manuscript: AR. Critical revision of the manuscript for important intellectual content: EK. Statistical analysis: AR and CP. Administrative, technical, or material support: CP. Supervision: EK.

## Endnotes


^[1]^ TPA is provided by the governmental agency for territorial cohesion, a branch of the Ministry of Finances; the service tracks regional revenues and expenditure fluxes collected and paid at the central and local levels.

